# A Gestalt account of human behavior is supported by evidence from switching between single and dual actions

**DOI:** 10.1038/s41598-023-47788-0

**Published:** 2023-12-01

**Authors:** Lynn Huestegge, Aleks Pieczykolan, Iring Koch

**Affiliations:** 1 University of Wuerzburg, Wuerzburg, Germany; 2 RFH Cologne, Cologne, Germany; 3 RWTH Aachen University, Aachen, Germany

**Keywords:** Human behaviour, Cognitive neuroscience, Learning and memory

## Abstract

The question of how behavior is represented in the mind lies at the core of psychology as the science of mind and behavior. While a long-standing research tradition has established two opposing fundamental views of *perceptual* representation, Structuralism and Gestalt psychology, we test both accounts with respect to *action* representation: Are multiple actions (characterizing human behavior in general) represented as the sum of their component actions (Structuralist view) or holistically (Gestalt view)? Using a single-/dual-response switch paradigm, we analyzed switches between dual ([A + B]) and single ([A], [B]) responses across different effector systems and revealed comparable performance in partial repetitions and full switches of behavioral requirements (e.g., in [A + B] → [A] vs. [B] → [A], or [A] → [A + B] vs. [B] → [A + B]), but only when the presence of dimensional overlap between responses allows for Gestalt formation. This evidence for a Gestalt view of behavior in our paradigm challenges some fundamental assumptions in current (tacitly Structuralist) action control theories (in particular the idea that all actions are represented compositionally with reference to their components), provides a novel explanatory angle for understanding complex, highly synchronized human behavior (e.g., dance), and delimitates the degree to which complex behavior can be analyzed in terms of its basic components.

## Introduction

While a substantial body of research on cognition has been established regarding mechanisms underlying action control^1^, the basic issue of how behavior—typically characterized by multiple, cross-modal motor movements—is mentally represented has not yet received sufficient attention. For example, while driving a car, is pushing the clutch (pedal movement) and shifting the gear (manual movement) represented as a single action or just as the sum of two behavioral parts? This conceptual neglect is particularly surprising considering a long-standing research tradition with respect to perceptual representations. That is, on the input (perceptual) side of processing, two opposing general views have been historically established as predecessors of modern information processing approaches: Structuralism and Gestalt psychology. While Structuralism (harking back to Wundt and Titchener^2,3^) assumes that complex mental representations can be analyzed in terms of their (atom-like) components, Gestalt psychology in the Wertheimer^4^ tradition rather proposes that a complex, holistic representation (“the whole”) is *different from* the sum of its parts^5^. Examples for Gestalt effects abound: In the domain of perception, it was shown that holistic figures can emerge from seemingly unrelated, simultaneously processed stimulus elements (points, lines …). Relatedly, in the domain of learning there is a long-standing debate on elemental versus configural *stimulus* learning in association formation^6,7^. However, while both Structuralists and Gestalt psychologists were also concerned with studying and conceptualizing human behavior, there has been a surprising scarcity of research on the specific question of how simultaneously processed actions (essentially characterizing *all* real-life human behavior) are mentally represented: As the sums of their elementary behavioral parts or as distinct action Gestalten?

A typical method to study cognitive representations and their dynamics is sequential performance analysis. The underlying assumption is that changes in cognitive representations yield performance costs compared to situations involving unchanged (or shared) cognitive representations, the latter often enabling relative performance (repetition) benefits (also referred to as priming). For example, performance declines when subjects change from one action representation to another (e.g., from left to right key press^8^), or from one task representation to another (task switching^9^). In this way, performance can—under otherwise controlled conditions—serve as an empirical marker for shared versus different underlying cognitive representations between successive actions.

Here, we utilized this rationale to test whether (simultaneous) multiple actions share (or do not share) mental representations with their constituent component actions by having participants switch between single and dual responses from trial to trial (single-/dual-switch paradigm). This novel paradigm is a combined derivative of both the dual-task and the task-switching paradigm^9,10^. Unlike these previous paradigms, we did not focus on comparing single- versus dual-task performance or on differences between switching versus repeating tasks, but more specifically on sequential transitions across single- and dual-responses.

Overall, we expected repeated action requirements (single → single, dual → dual) to yield performance benefits, serving as a proof of concept for the underlying assumption that unchanged cognitive representations result in (relative) benefits. However, the Gestalt and Structuralist accounts of dual-action representation fundamentally differ with respect to predictions for action type switches, thereby allowing (probably for the first time) for a rigorous *experimentum crucis* (Table 1): If the Structuralist account is true, dual responses (e.g., simultaneous processing of responses [A + B]) should share cognitive representations with either component response ([A], [B]), and thus a switch from [A + B] to [A] should result in better performance than a switch from [B] to [A] (*partial repetition benefit* for *single* responses). Similarly, the A-part of [A + B] should be performed better when preceded by [A] instead of [B] (*partial repetition benefit* for *dual* responses).

**Table 1:** Hypothesized RT pattern in Trial N for the Structuralist (“A + B” = “A” + “B”) versus Gestalt (“A + B” = “C”) view of complex action as a function of trial sequence (N − 1 → N).

Relevant action (trial N: A, B)	Action condition (trial N: single, dual)	Trial N − 1	Trial N	Trial N sequence type	Structuralist view	Gestalt view
A	Single	A	A	Repetition	A fast	A fast
		**A + B**	**A**	**Partial Repetition**	**A fast**	**A slow**
		B	A	Switch	A slow	A slow
	Dual	A + B	A + B	Repetition (for A)	A fast	A fast
		**A**	**A + B**	**Partial Repetition (for A)**	**A fast**	**A slow**
		B	A + B	Switch (for A)	A slow	A slow
B	Single	B	B	Repetition	B fast	B fast
		**A + B**	**B**	**Partial Repetition**	**B fast**	**B slow**
		A	B	Switch	B slow	B slow
	Dual	A + B	A + B	Repetition (for B)	B fast	B fast
		**B**	**A + B**	**Partial Repetition (for B)**	**B fast**	**B slow**
		A	A + B	Switch (for B)	B slow	B slow

Predictions diverge for Partial Repetition conditions (bold).

In contrast, the Gestalt account assumes that [A + B] is represented as a distinct Gestalt (in the strong sense that it no longer resembles its components), similar to an unrelated (e.g., [C]) response. The Gestalt account thus predicts similar performance for switching from [A + B] to [A] as for switching from [B] to [A], and similar performance in the A-part of [A + B] when preceded by [A] versus [B] (lack of partial repetition benefits relative to switches). Note that this Gestalt account radically differs from existing (tacitly Structuralist) feature integration/binding accounts of action representation, which assume that the integrated whole is just “more than” (not essentially “different from”) the sum of its parts^11^: That is, an integration of [A] and [B] into [A + B] still retains a conceptual link to its structural components, so that, for example, the mental activation of a component is able to retrieve the whole integrated compound [A + B] (this integration account might also be referred to as a “weak” Gestalt account, see General Discussion). This is no longer possible within a “strong” Gestalt account relevant for the present study where the combination of [A] and [B] is represented *differently* (as [C]).

In fact, recent studies have indeed revealed evidence for partial repetition benefits in a paradigm involving switches between single and dual tasks (thereby indicating compositional, Structuralist representations^12,13^). However, the particular tasks in these studies were somewhat artificial in that they were deliberately designed to be maximally unrelated (in terms of the task characteristics involved: a spatial visual-manual task was combined with a non-spatial auditory-vocal task). In everyday behavior, however, performing *completely* unrelated tasks at a time rarely (if ever) occurs, as concurrent actions usually have a common target/goal or at least share some (e.g., spatial) characteristics, which consequently opens up the possibility of providing shared dimensions upon which the representation of action Gestalten can be based. Thus, we present a series of experiments designed to study trial-by-trial transition effects between single- and dual-response trials to differentiate between the Structuralist and Gestalt accounts of dual-action representation. Crucially, across experiments we systematically reduced the potential for Gestalt formation: While in Experiment 1A/B responses in dual-response trials are spatially compatible and triggered by a common stimulus, Experiment 2 involves separate stimuli. Experiment 3 additionally introduces spatially incompatible responses, while Experiment 4 finally removes any (spatial) dimensional overlap between responses to potentially withdraw any basis of Gestalt formation.

## Methods

### Overview of experiments

**Figure 1:**
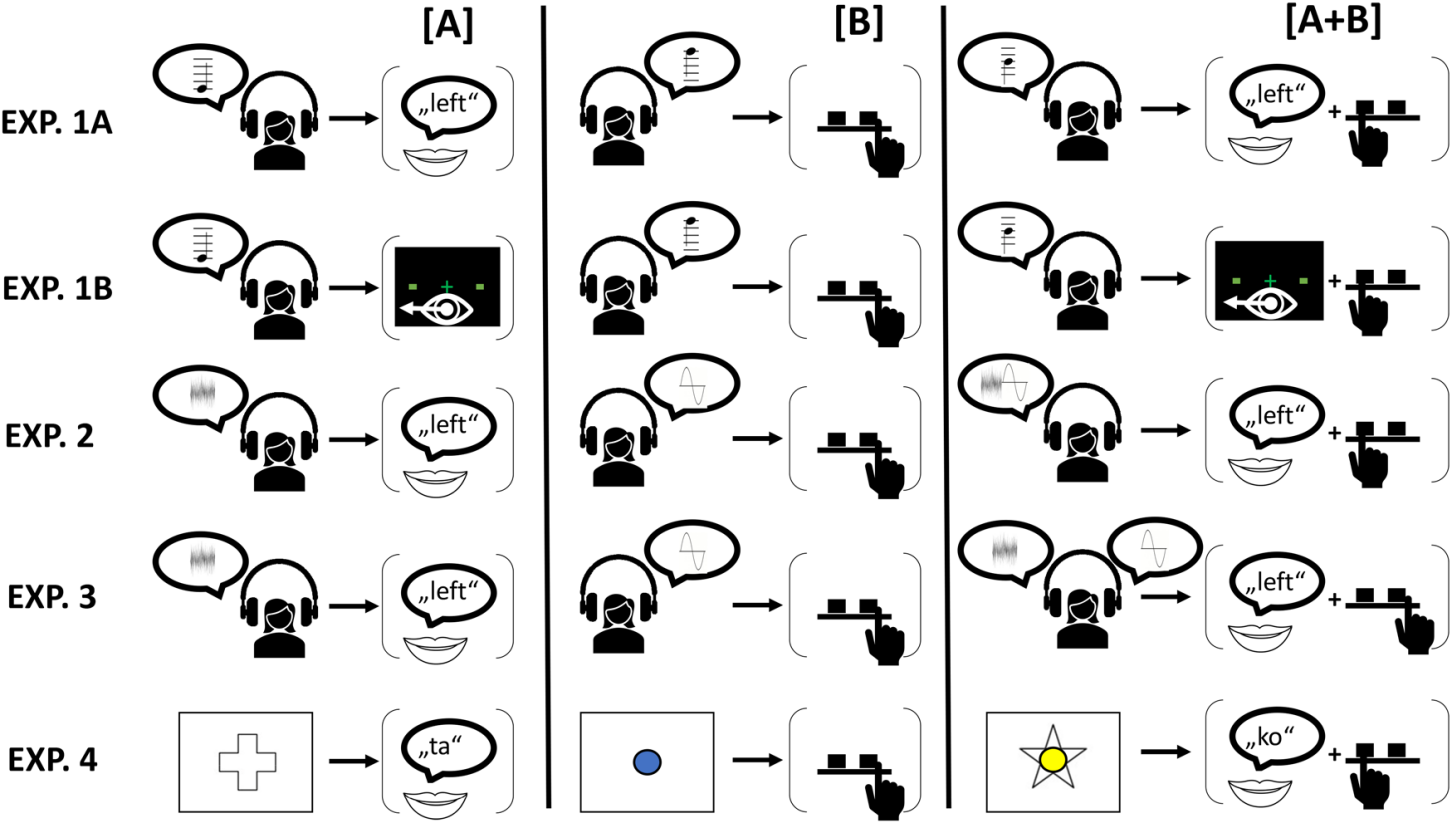
Stimuli and required responses (examples). Single action [A] refers to vocal response (Exp. 1B: oculomotor), single action [B] refers to manual response, dual action [A + B] refers to combined vocal (oculomotor) and manual response. Exp. 1–3 involved the display of a black screen with a central fixation cross throughout. Response condition (single [A], single [B], dual [A + B]) was indicated via tone pitch (low/medium/high) in Exp. 1, via lateralized tone type (noise/harmonic) in Exp. 2/3 (which could occur on different ears in dual trials only in Exp. 3), and via figure type (inner/outer) in Exp. 4. Assignments of stimulus categories (e.g., tone pitch) to response conditions were counterbalanced. Response direction was indicated via sound direction (left/right ear, Exp. 1–3) or via the shape of the outer visual figure (stimulus 1: plus/star) or the color (stimulus 2: blue/yellow) of the inner circle (Exp. 4).

The predictions from the two accounts with respect to action representation are evaluated across five experiments, with decreasing potential for action Gestalt formation. Experiment 1 involves *single* (auditory) stimuli triggering either one or two (spatially *compatible*) responses (see Fig. 1 for a visualization of all experiments). To avoid a potentially tight a priori coordination of component responses within a single effector system^14^ and thus to test our hypotheses with maximal rigor, we always combined responses across different systems throughout (Experiments 1A, 2, 3, 4: manual-vocal, Experiment 1B: manual-oculomotor). Thus, for example, in Experiment 1A participants switched between performing a single (left or right) manual response ([A]), a single vocal response ([B]: saying “left” or “right”), and a manual-vocal dual response ([A + B]: both always in same direction). To study potential limits of forming Gestalt representations, the following experiments systematically introduced elements that could be assumed to promote Structuralist representations (Table 2). Experiment 1B combined manual responses with oculomotor (instead of vocal) responses, as one might argue that it is more difficult to form action Gestalten with an effector system that is usually considered to be controlled via rather distinct, more encapsulated motor networks^15^. Experiment 2 involved *distinct stimuli* for both (manual & vocal) responses (which nevertheless are still always spatially compatible in dual-response trials) to test whether distinct stimulation of the two responses might prevent Gestalt formation. Experiment 3 additionally introduced spatially *incompatible* dual responses, thereby addressing the presence/absence of a constant “common fate”-like principle as a potential prerequisite of Gestalt formation. As in Experiment 3 the identity of one response does no longer allow participants to infer the identity of the other response in dual-response conditions (if one response is “right”, the other response might be “left” *or* “right”, depending on the respective stimulus), this qualifies as a rather typical dual-task experiment. Finally, Experiment 4 is similar to Experiment 3 but any (spatial) dimensional overlap between dual responses was removed (e.g., vocal response “ta” instead of “right”), which should effectively withdraw the necessary basis of Gestalt formation (thus requiring Structuralist representations as reflected in partial repetition benefits similar to those observed in^12,13^, see Fig. 1).

**Table 2:** Overview of experiments and their presumable potential to promote motor Gestalt formation based on task characteristics.

Experiment	Factors potentially promoting Gestalt formation		
	Compound stimulation (same stimulus dimension carries information for both actions)	Compound responding (both actions always same spatial direction)	Cross-response dimensional overlap (both actions spatial)
1a (Manual-Vocal)	X	X	X
1b (Manual-Saccade)^a^	X	X	X
2 (Manual-Vocal)	–	X	X
3 (Manual-Vocal)	–	–	X
4 (Manual-Vocal)	–	–	–

^a^Manual-Saccade effector combination potentially less conducive to Gestalt formation (than Manual-Vocal in Exp. 1a).

### Participants

Ninety-six participants were tested. Power analyses suggested that demonstrating partial repetition benefits in young adults^12^ at 1-β = .90 requires only 6 (dual-response trials) or 11 (single-response trials) participants. Eighteen participants with normal or corrected-to normal vision took part in Experiments 1–3 (Exp. 1A: 13 females, mean age: 23 years, *SD* = 2.9; Exp. 1B: 14 females, mean age: 24 years, *SD* = 3.6; Exp. 2: 14 females, mean age: 23 years, *SD* = 5.0; Exp. 3: 15 females, mean age: 21 years, *SD* = 2.1). Experiment 4 involved 24 participants (22 females, mean age = 21 years, *SD* = 2.6). All gave informed consent and received course credits for participation. All experiments were performed in accordance with relevant guidelines and regulations (including the Declaration of Helsinki). The experimental protocols used in the present study were approved by a vote from the ethics committee of the Institute of Psychology at University of Wuerzburg.

### Apparatus and stimuli

Participants were seated 65 cm in front of a 21″ cathode ray monitor (temporal resolution: 100 Hz; spatial resolution: 1,024 × 768 pixels) and a microphone for vocal response recordings (Experiments 1A, 2, 3, 4). A chinrest was used to minimize head movements. Eye movements were recorded in all experiments, either because they represented one of the two instructed responses (Exp. 1B) or to control for unwanted eye movements when these were not explicit part of the task (as saccades would represent additional actions which are well-known to potentially interfere with other, concurrent actions^15^, even when these eye movements occur involuntarily^16^). In Exp. 1–2, a green central fixation cross (0.9°) was displayed on the screen (on black background) together with two green saccade targets (squares of 0.9° side length) at 8.25° eccentricity to the left and right (serving as eye movement targets in Exp. 1B). These peripheral targets were not present in Exp. 3–4. Participants wore headphones for auditory stimulus presentation in Exp. 1–3 (Exp. 1: tones on the left/right ear using three frequencies to indicate single-/dual-responses, Exp. 2–3: left/right pink noise or harmonic tones, with tone type indicating manual/vocal responses). Experiment 4 involved the presence of visual symbols (“plus”/”star”/none) at the screen center to indicate vocal responses (uttering “ta”/”ko”/nothing), and an additional central colored circle (blue/yellow/no color) to indicate manual responses (left/right/no key press).

Eye movements of the right eye were measured using an EyeLink 1000 infrared reflection system (SR Research, Osgoode, Ontario, Canada) with a temporal resolution of 500 Hz. Experiments were programmed using Experiment Builder (SR Research, Osgoode, Ontario, Canada). The space bar of a keyboard located in front of the participant was operated during eye tracker calibrations (prior to each block). Two keys on the keyboard (left Ctrl and right arrow in Exp. 1–3, left/right arrow in Exp. 4) were chosen as manual response keys operated by the left/right index finger, respectively.

### Procedure

Response types (single [A], single [B], dual [A + B]) were randomly distributed within blocks of trials. In Experiment 1, the pitch of a lateralized tone indicated whether participants should perform a single manual response [A], a single vocal/oculomotor response [B] (in Exp. 1A/B, respectively), or both [A + B]^17^. The combination of manual and vocal responses is part and parcel of daily communication and typical for research in the field of multitasking^10^, and a previous study^18^ reported evidence for tendencies to synchronize speech and manual action in a “Gestalt-like” manner across these effector systems. In addition, we also considered using oculomotor responses as they are assumed to be highly distinct from other effector systems, and—given that both movements are not coordinated towards a common target as, for example, in eye-hand coordination—probably even less likely to be represented in a Gestalt-like fashion together with manual actions^15^. Thus, Experiment 1B serves as a test whether Gestalt representations generalize across various effector system pairings.

Each trial started with the 50 ms presentation of the auditory stimulus (or the visual stimulus in Exp. 4, which was present throughout the trial). In Experiment 1, the pitch of the auditory stimulus indicated the response condition (e.g.: 200 Hz: [A], 600 Hz: [A + B], 3200 Hz: [B], mapping counterbalanced across participants). The auditory stimuli were presented either on the left or right ear, indicating the required response direction (left/right key press, left/right eye movement, uttering the words “left”/”right”). The inter-stimulus interval (within which the response(s) had to be initiated) was held constant (3200 ms, plus an additional 200 ms in Experiment 4 to ensure a visual separation between stimuli across trials).

As using three different stimuli for the three response conditions in Experiment 1 may promote a more separate (Gestalt-like) cognitive representation of the dual-response condition, we decided to use two separate stimuli for dual conditions in Experiment 2. Specifically, Experiment 2 involved two different (easily distinguishable) lateralized sound types (pink noise vs. harmonic tone) presented at the same time to trigger (vocal vs. manual) responses (sound type-effector mapping counterbalanced across participants). However, responses in dual-response trials were still spatially compatible throughout (the two sounds always occurred on the same side in dual-response trials), thereby potentially promoting Gestalt representations.

In contrast, Experiment 3 also involved dual-response trials requiring spatially incompatible responses, since both sounds (in dual-response conditions) could (unpredictably) occur on the same side as well as on different sides. Thus, Experiment 3 represents a rather typical dual-task study. Finally, Experiment 4 was similar to Experiment 3 but (spatial) dimensional overlap between responses was removed by introducing vocal responses without any spatial characteristics (uttering “ta”/“ko” to central visual “plus sign”/“star” symbols, respectively). Thus, Experiment 4 is conceptually similar to previous studies on unrelated dual tasks in which partial repetition benefits were found^12,13^. The lack of both symbols indicates that no vocal response is needed (manual single response). Manual responses were spatial (left/right) key presses operated by the index finger of the right hand (which otherwise rested in between both response keys), and were triggered by a centrally presented color (in a circle, e.g., blue for left, yellow for right, absence of color indicating no manual response).

Participants were generally asked to respond as fast and accurately as possible. There was no priority or order instruction with respect to the individual responses in dual-response trials. Experiments 1–2 consisted of 540 trials in total, divided into five blocks. Experiment 3 involved 8 blocks (108 trials each), Experiment 4 involved 7 blocks (96 trials each). Response conditions ([A], [B], [A + B]) occurred equally often.

### Design

In all five experiments, the independent variables were response (manual vs. vocal/oculomotor), response condition (single, dual), and transition, the latter comprising three crucial categories: full repetition (single condition: [A] → [A], [B] → [B]; dual condition: [A + B] → [A + B]), partial repetition (single: [A + B] → [A], [A + B] → [B], dual condition: [A] → [A + B], [B] → [A + B]), and switches (single condition: [A] → [B], [B] → [A], dual condition: [A] → [A + B], [B] → [A + B] (note that the relevant part of the complex action which may or may not benefit from partial repetitions in the given examples—e.g., assuming the validity of the Structuralist account—is underlined).

## Results and discussion

In all experiments that did not require eye movements as an instructed response, trials involving the execution of eye movements > 2° were not considered in RT analyses as these may compromise the interpretation of RT effects in the other effector system domains (19.1%, 9.3%, 16.4%, 13.1% of trials in Experiment 1A, 2, 3, 4). Errors were defined as trials in which the required response(s) was/were not executed. RTs were only considered in error-free trials (RTs < 150 ms (saccade RTs: < 70 ms) and RTs > 2000 ms were discarded). To address response transition effects, RTs were only analyzed when both the current and the previous trial were error-free.

**Figure 2:**
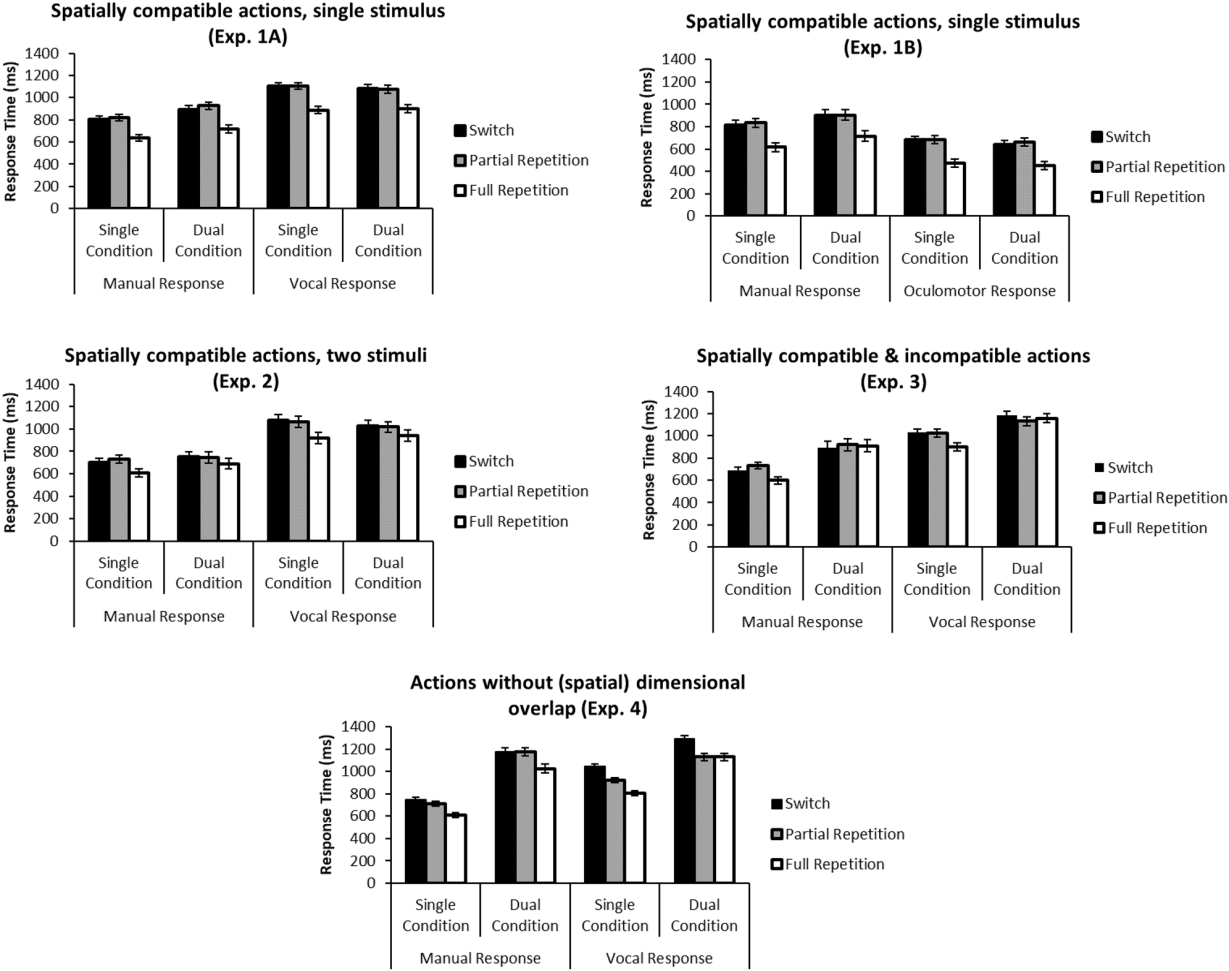
Response time results (error bars represent SE). Full repetitions (white bars) are always associated with fastest responses. Partial repetition benefits (difference between black and grey bars) only emerge for vocal responses in Experiment 4 (i.e., in the absence of spatial dimensional overlap across the two actions).

There were significant main effects of transition on RTs in all experiments (Exp. 1A: *F*(2,34) = 50.29, *p* < .001, *η*_*p*_^2^ = .863, Exp. 1B: *F*(2,34) = 123.56, *p* < .001, *η*_*p*_^2^ = .879, Exp. 2: *F*(2,34) = 62.74, *p* < .001, *η*_*p*_^2^ = .787, Exp. 3: *F*(2,32) = 15.20, *p* < .001, *η*_*p*_^2^ = .487, Exp. 4: *F*(2,46) = 79.94, *p* < .001, *η*_*p*_^2^ = .777). Figure 2 demonstrates that full repetitions (e.g., executing a single manual response after a single manual response) were executed faster than both partial repetitions and switches (*p* ≤ .001 for all post hoc LSD contrasts) across all experiments (1A, 1B, 2, 3, 4), serving as a proof of concept for the main assumption that unchanged cognitive representations result in (relative) benefits.

Most importantly, RTs did not significantly differ between partial repetition and switch conditions in Experiments 1–3 (*p* > .12 for all post hoc LSD contrasts, see Table 3 for a complete overview of ANOVA results). That is, there was no statistical evidence whatsoever for a partial repetition benefit in any of these experiments involving spatial dimensional overlap between responses across effector systems (see Fig. 2). Also note that the maximum border of the 95% *CI* for the partial repetition benefit amounted to 11 ms, 3 ms, 12 ms, and 15 ms (for Experiments 1A, 1B, 2, and 3, respectively), which is clearly too small to be compatible with the assumption of a meaningful partial repetition benefit overall (especially when compared to the size of the full repetition benefit, see Fig. 2). Specifically, the maximum border of the 95% *CIs* (see above) never includes an (absolute) effect size of 36 ms that was reported in a previous study for a partial repetition benefit in single task trials, or of 18/62 ms that were previously reported as partial repetition benefits for dual-task trials^12^, serving as a smallest effect size of interest. Therefore, in accordance with typical procedures recommended for equivalence testing^19^, we can conclude that there is significant statistical evidence for the *absence* of a meaningful partial repetition benefit effect throughout Experiments 1–3.

**Table 3:** ANOVA results.

Experiment	Factor/interaction	Dependent Variable	df	F	*p*	η_p_^2^
Exp. 1A	Response (manual, vocal)	RT (ms)	1, 17	49.14	< .001	.743
		Error rate (%)	1, 17	0.43	.521	.025
	Response Condition (single, dual)	RT (ms)	1, 17	4.92	.040	.225
		Error rate (%)	1, 17	5.21	.036	.234
	Transition (full repetition, partial repetition, switch) Pairwise Contrasts (LSD adjusted)	RT (ms) (incl./excl. spatial priming)	2, 34	50.29/67.00	< .001/ < .001	.863/.798
		Contrast full-partial			< .001/ < .001	
		Contrast full-switch			< .001/ < .001	
		Contrast partial-switch			.368/.400	
		Error rate (%)	2, 34	4.63	.017	.214
		Contrast full-partial			.024	
		Contrast full-switch			.627	
		Contrast partial-switch			.027	
	Response*Response Condition	RT (ms)	1, 17	6.31	.022	.271
		Error rate (%)	1, 17	4.01	.061	.191
	Response*Transition	RT (ms)	2, 34	0.55	.588	.064
		Error rate (%)	2, 34	2.70	.081	.137
	Response Condition*Transition	RT (ms)	2, 34	0.19	.832	.023
		Error rate (%)	2, 34	5.53	.008	.246
	Response*Response Condition*Transition	RT (ms)	2, 34	1.70	.214	.175
		Error rate (%)	2, 34	4.55	.018	.211
Exp. 1B	Response (manual, oculomotor)	RT (ms)	1, 17	116.66	< .001	.873
		Error rate (%)	1, 17	5.54	.031	.246
	Response Condition (single, dual)	RT (ms)	1, 17	1.51	.236	.082
		Error rate (%)	1, 17	0.18	.676	.011
	Transition (full repetition, partial repetition, switch) Pairwise Contrasts (LSD adjusted)	RT (ms) (incl./excl. spatial priming)	2, 34	123.56/125.27	< .001/ < .001	.879/.881
		Contrast full-partial			< .001/ < .001	
		Contrast full-switch			< .001/ < .001	
		Contrast partial-switch			.123/.688	
		Error rate (%)	2, 34	10.41	< .001	.380
		Contrast full-partial			.002	
		Contrast full-switch			.683	
		Contrast partial-switch			.001	
	Response*Response Condition	RT (ms)	1, 17	6.98	.017	.291
		Error rate (%)	1, 17	0.11	.745	.006
	Response*Transition	RT (ms)	2, 34	0.05	.954	.003
		Error rate (%)	2, 34	4.73	.015	.218
	Response Condition*Transition	RT (ms)	2, 34	0.57	.573	.032
		Error rate (%)	2, 34	4.70	.016	.217
	Response*Response Condition*Transition	RT (ms)	2, 34	0.99	.382	.055
		Error rate (%)	2, 34	0.95	.398	.053
Exp. 2	Response (manual, vocal)	RT (ms)	1, 17	166.61	< .001	.907
		Error rate (%)	1, 17	31.91	< .001	.652
	Response Condition (single, dual)	RT (ms)	1, 17	0.58	.457	.033
		Error rate (%)	1, 17	1.27	.275	.069
	Transition (full repetition, partial repetition, switch) Pairwise Contrasts (LSD adjusted)	RT (ms) (incl./excl. spatial priming)	2, 34	62.74/29.32	< .001/ < .001	.787/.633
		Contrast full-partial			< .001/ < .001	
		Contrast full-switch			< .001/ < .001	
		Contrast partial-switch			.934/.597	
		Error rate (%)	2, 34	12.82	< .001	.430
		Contrast full-partial			.002	
		Contrast full-switch			< .001	
		Contrast partial-switch			.589	
	Response*Response Condition	RT (ms)	1, 17	6.22	.023	.268
		Error rate (%)	1, 17	0.68	.422	.038
	Response*Transition	RT (ms)	2, 34	2.22	.124	.116
		Error rate (%)	2, 34	14.82	< .001	.466
	Response Condition*Transition	RT (ms)	2, 34	11.46	.001	.403
		Error rate (%)	2, 34	3.52	.041	.172
	Response*Response Condition*Transition	RT (ms)	2, 34	1.68	.209	.090
		Error rate (%)	2, 34	4.75	.015	.218
Experiment	Factor/Interaction	Dependent Variable	df	F	p	η_p_^2^
Exp. 3	Response (manual, vocal)	RT (ms)	1, 16	123.53	< .001	.885
		Error rate (%)	1, 17	5.20	.036	.234
	Response Condition (single, dual)	RT (ms)	1, 16	51.24	< .001	.762
		Error rate (%)	1, 17	23.46	< .001	.580
	Transition (full repetition, partial repetition, switch) Pairwise Contrasts (LSD adjusted)	RT (ms) (incl./excl. spatial priming)	2, 32	15.20/5.30	< .001/.016	.487/.249
		Contrast full-partial			< .001/.015	
		Contrast full-switch			.001/.033	
		Contrast partial-switch			.704/.704	
		Error rate (%)	2, 34	1.92	.162	.102
		Contrast full-partial			.864	
		Contrast full-switch			.068	
		Contrast partial-switch			.101	
	Response*Response Condition	RT (ms)	1, 16	2.87	.110	.152
		Error rate (%)	1, 17	1.45	.245	.079
	Response*Transition	RT (ms)	2, 32	7.92	.005	.331
		Error rate (%)	2, 34	3.63	.039	.176
	Response Condition*Transition	RT (ms)	2, 32	14.43	.001	.474
		Error rate (%)	2, 34	6.62	.004	.280
	Response*Response Condition*Transition	RT (ms)	2, 32	0.50	.589	.030
		Error rate (%)	2, 34	4.06	.031	.193
Exp. 4	Response (manual, vocal)	RT (ms)	1, 23	48.90	< .001	.680
		Error rate (%)	1, 23	8.06	.009	.260
	Response Condition (single, dual)	RT (ms)	1, 23	331.99	< .001	.935
		Error rate (%)	1, 23	13.54	.001	.371
	Transition (full repetition, partial repetition, switch) Pairwise Contrasts (LSD adjusted)	RT (ms) (incl./excl. spatial priming)	2, 46	79.94/46.18	< .001/ < .001	.777/.668
		Contrast full-partial			< .001/ < .001	
		Contrast full-switch			< .001/ < .001	
		Contrast partial-switch			< .001/ < .001	
		Error rate (%)	2, 46	16.68	< .001	.420
		Contrast full-partial			.454/.002	
		Contrast full-switch			< .001/.004	
		Contrast partial-switch			.001/.004	
	Response*Response Condition	RT (ms)	1, 23	35.34	< .001	.606
		Error rate (%)	1, 23	3.19	.088	.122
	Response*Transition	RT (ms)	2, 46	40.37	< .001	.637
		Error rate (%)	2, 46	< 1		
	Response Condition*Transition	RT (ms)	2, 46	15.73	< .001	.406
		Error rate (%)	2, 46	4.29	.022	.157
	Response*Response Condition*Transition	RT (ms)	2, 46	16.80	< .001	.422
		Error rate (%)	2, 46	4.23	.028	.155

In Experiment 2, data from the first block of one participant had to be excluded due to technical difficulties. One participant in Exp. 3 did not contribute sufficient valid RT data for the trial sequence analyses (resulting in lower df). Transition effects are reported for trials including spatial priming trials (e.g., left → left) and for trials excluding spatial priming (trials not involving repeated response direction).

Thus, even though in Experiment 2 separate stimuli were used to trigger both responses (based on the idea that separate stimulation of two responses might counteract Gestalt formation), we still observed evidence for Gestalt representations. Likewise, even though in Experiment 3 the two spatial responses were spatially incompatible in half of the dual-response trials (based on the idea that the presence of spatially incompatible responses might prevent Gestalt formation), these situations also evidently still enabled Gestalt representations.

Importantly, however, in Experiment 4, where we completely removed any spatial dimensional overlap between responses, the data pattern was fundamentally different: Here, a statistically robust overall partial repetition effect (95% CI 26–79 ms overall, see Fig. 2) emerged for the vocal dual-task condition, indicating the lack of robust Gestalt formation in the absence of a common (spatial) dimension for the two component responses in Experiment 4. Note though that we did not find this similarly for the manual dual-task condition, where a deviant pattern in RTs was observed, as indicated by the corresponding interactions. The fact that these partial repetition benefits were present in the vocal (but not in the manual) responses might be due to the fact that in manual-vocal dual tasks, vocal response processing is typically prioritized^20,21^ and might thus benefit more from repeated (component) responses. Note that this finding of partial repetition benefits in Experiment 4 is further supported by two other recent studies that analyzed switches between single- and dual-task trials in situations also characterized by low (spatial) cross-task dimensional overlap^12,13^. Together, this is very robust empirical evidence for compositional, Structuralist action representation when (via the *absence* of dimensional overlap) a crucial precondition of action Gestalt formation is removed.

Finally, we additionally re-analyzed the RT data by excluding all trials involving the successive execution of exactly the same response requirements (full stimulus repetitions involving response direction repetitions such as left → left), as one might argue that repetition priming (due to repeating the exact same stimulus–response episode) might distort the results. However, this did not substantially alter the pattern of transition effects (see Table 3), indicating that the findings were not solely driven by specific identity priming mechanisms. In addition, this observation empirically supports our tenet that the relevant response components here are defined by effector modalities (e.g., [A] refers to manual, [B] to vocal), not by response direction (see also^22^). Note that we also did not find any evidence for partial repetition benefits when only analyzing consecutive trials involving the *same* spatial response direction in Experiments 1–2, where a sufficient number of corresponding trials was available. Finally, refraining from the exclusion of trials with (uninstructed) saccade execution (in Experiments 1A, 2, 3, 4) also did not change the overall pattern of results.

Note that the interpretation of the main effect of transition on RTs is not severely compromised by any interaction of transition with other factors: In Experiment 2, a significant interaction with response condition indicated that the full repetition advantage is more pronounced in single versus dual conditions, whereas in single-task conditions of Experiment 3 a partial repetition cost in manual RTs was traded off against a partial repetition benefit in vocal error rates (no partial repetition benefit or cost was present in RTs or error rates in the dual-task conditions of Exp. 3). In sum, the lack of partial repetition benefits (e.g., better performance of [A] when preceded by [A + B] instead of [B], or better performance of the A-part of [A + B] when preceded by [A] instead of [B]) in Experiments 1–3 shows that dual responses ([A + B]) are distinctly represented without reference to their constituent component responses ([A], [B]), supporting the assumption of Gestalt representations of simultaneous multiple responses whenever (here: spatial) dimensional overlap^23^ is present as a reference for establishing a Gestalt representation.

One interesting observation is that in Experiment 3 we did not find clear evidence for a full repetition benefit in the dual-task data (see Fig. 2). One possible explanation would be that due to the presence of both spatially compatible and incompatible dual-task trials in this experiment, participants adopted a strategy within which they no longer made use of the (principally available) benefit of a full repetition, as the incompatible trials were experienced as so difficult to process that they started the processing of any dual-task trial “from scratch”. While this mechanism is rather speculative, it is important to note that any such (potentially strategic) mechanism underlying this particular aspect of the data pattern would not endanger our general conclusions regarding the underlying representation formats throughout the experiments of the present study.

While overall error rates are probably too low for meaningful interpretation in some experiments (participants responded correctly in more than 96.5% of the trials in each of the Experiments 1–2), statistical analyses revealed no evidence for partial repetition benefits across Experiments 1–3. If anything, we observed slightly *higher* error rates in partial repetition versus switch conditions in the dual manual conditions of Experiment 1A/B and in the single manual condition in Experiment 1B (*ps* < .05 for post hoc LSD contrasts). However, this was not a consistent pattern, since it did neither occur in any of the nine remaining comparisons (see Table 4), nor anywhere in the RT data. Unlike in Experiments 1–3, however, we observed substantial evidence for partial repetition benefits in the error rates of Experiment 4 (in both single- and dual-task conditions). In sum, this confirms the RT-based conclusions that dual responses are represented in a Structuralist fashion in the absence of dimensional overlap.

**Table 4:** Error rates.

Exp	Manual response						Vocal/oculomotor response					
	Single condition			Dual condition			Single condition			Dual condition		
	Switch	Partial repetition	Full repetition	Switch	Partial repetition	Full repetition	Switch	Partial repetition	Full repetition	Switch	Partial repetition	Full repetition
1A	2.4 (0.6)	2.3 (0.7)	1.5 (0.8)	3.6 (1.2)	7.1 (1.5)	5.8 (1.3)	3.7 (1.1)	4.3 (1.3)	1.7 (0.6)	3.1 (0.6)	3.2 (1.2)	3.2 (1.1)
1B	3.9 (1.8)	6.4 (1.7)	1.2 (0.4)	1.4 (0.6)	5.8 (2.3)	3.9 (1.0)	2.8 (0.9)	3.8 (0.8)	1.7 (0.6)	1.9 (0.5)	2.1 (0.8)	2.2 (0.6)
2	0.2 (0.1)	0.0 (0.0)	0.3 (0.2)	0.0 (0.0)	0.0 (0.0)	0.0 (0.0)	5.3 (1.1)	6.1 (0.9)	1.6 (0.4)	4.2 (1.4)	3.1 (0.9)	2.1 (1.3)
3	2.6 (1.1)	1.5 (0.3)	1.3 (0.8)	22.0 (3.5)	19.6 (3.4)	22.5 (3.4)	6.9 (1.1)	4.5 (0.9)	2.4 (0.6)	27.9 (6.1)	30.4 (6.5)	29.4 (6.4)
4	4.3 (0.7)	2.0 (0.4)	1.7 (0.4)	5.7 (0.9)	3.9 (0.8)	4.4 (0.8)	2.5 (0.6)	2.3 (0.5)	0.8 (0.3)	4.4 (0.7)	1.6 (0.4)	2.4 (0.5)

SE in parentheses. Due to hand position shifts some participants temporarily pressed different (e.g., adjacent) keys than required. As this did not meaningfully change the task, these data were corrected and retained in the analyses.

## General discussion

A Gestalt view of human action, which was reliably supported by all relevant contrasts in Experiments 1A, 1B, 2, and 3, has important implications for current action control theories. Traditionally, the field in which cognitive underpinnings of multiple action control are discussed is multitasking research. Interestingly, however, the question of Gestalt versus Structuralist representations of behavior has never been a vital empirical or conceptual concern in this field.

Early theorizing has sometimes interpreted the emergence of dual-task interference per se as evidence for the claim that multitasking (which essentially characterizes any human real-life action in general) is more than the sum of its component tasks^24^. However, this view never entailed the more radical idea that dual-action representations may not resemble their constituent components at all. Instead, more recent theories explicitly^11^ or tacitly assume that the cognitive representation of a component task always remains structurally comparable under single- and dual-task requirements. Specifically, performance decrements elicited by the presence of additional action requirements are assumed to originate from interrupted (generic bottleneck models^10^) or strategically deferred^25^ component task processing, or because resource competition, crosstalk phenomena, or activation/inhibition dynamics between representations (associated with each component action) slow down component task processing^26–30^.

However, our present results challenge the underlying assumption of structurally comparable task/action representations under single and dual conditions as a universal, generally valid principle, an assumption that is also a prerequisite for the explanation of dual-task/dual-response costs in terms of the impact of secondary task presence on task processing. Instead, a Gestalt view would rather assume that complex action (i.e., action composed of at least two distinguishable sub-units) can—under appropriate conditions—be configured in a holistic way (similar to the notion of chunking in memory^31^), and thus attribute putative dual-task costs to a more complex (but essentially unitary) configuration (or selection) process associated with multiple action control. While action components here were defined in terms of effector modalities (e.g., [A] = saccade), a tenet that was also supported by the data, it may be worthwhile in future research to additionally focus on simple (instead of choice) responses.

The idea of action Gestalten being characterized by the lack of *any* strong reference to representations of their action components (“*different from* the sum of parts”) renders this view fundamentally distinct from previous action *integration* (or feature binding) accounts^11^, according to which complex action is merely “*more than* the sum” (this may also be referred to as a “weak” as opposed to a “strong” Gestalt account). Thus, in integration accounts the component representations still remain intact but are coded in a strongly associated manner^32–34^. Effects indicating integrated action (or task) representations were reported, for example, in studies on bi-manual control, task switching, and implicit learning^35–40^. A typical example for an integration account of multiple action control is a recent dual-task control framework^30^ in which each component response relevant in a dual-task setting is conceptualized as being bound to an integrated event file (containing information associated with the particular action), and the dynamic activation/inhibition patterns within and between event file representations eventually determine multiple action performance. Despite the idea of integrated representations within such an event file (consisting of stimuli, responses, effects etc.), this account still represents an essentially Structuralist (compositional) theorizing because each component response calls for a distinct event file. Despite this, this account is principally open to a possible integration of event files (or task representations, see also^37^).

Interestingly, according to some of these Structuralist integration accounts, most notably so-called feature binding accounts^41,42^, partial repetitions of features (here, on a conceptually somewhat higher level, referred to as task demands) should not yield performance benefits, but rather partial *mismatch costs*: As any partial repetition necessarily also implies some degree of change (in stimuli, context, or particular response requirements), this change (via the retrieval of an unwarranted type of task/action representation) can eventually make it *harder* to retrieve the appropriate action (see^41^, for empirical examples). Note, however, that this logic cannot be easily transferred to our study that involved switches between single and multiple actions, and we clearly did not find any substantial evidence for consistent performance costs associated with switching from dual to single actions (or vice versa) in our data. In a similar vein, some previous task switching studies addressed the related question of whether it is possible to re-use some control settings after a partial (vs. full) task switch, or whether all control settings need to be re-set (the latter being more in line with the assumption of holistic task representations). However, these studies yielded inconsistent results^43–47^, most likely due to the lack of a performance baseline regarding the component tasks (i.e., a baseline equivalent to the crucial single-response conditions in the present study).

Several studies in the realm of motor control have previously referred to Gestalt principles, but without empirically testing Structuralist against Gestalt predictions directly with respect to action representations. Nevertheless, these studies laid the groundwork for the present research. For example, in a pioneering attempt to re-conceptualize previous motor control findings in terms of Gestalt principles, Klapp and Jagacinski^48^ interpreted the observation that choice reaction time depends on motor chunk complexity as resulting from a motor Gestalt that must be programmed prior to any of its component gestures. However, they did not experimentally rule out the alternative (Structuralist) explanation that a motor chunk might still be represented in terms of its components. Another study^49^ reported that lateral oscillations of two index fingers are easier to synchronize when the movements are symmetric on a perceptual level (not on the level of homologous muscles). However, this effect basically demonstrates that perceptual Gestalt principles can be utilized to guide motor control, but it does not directly address the nature of action representations per se. Taken together, the essential question of whether mental representations of multiple actions can be organized in terms of motor Gestalten has not been sufficiently addressed in prior research.

Gestalt psychology has often been criticized for exhibiting a lack of clear, quantifiable predictions and for assuming rather opaque underlying mechanisms^5^. This issue may have prevented a more substantial generalization to other domains, including motor control. The present study demonstrates that—by developing a novel single-/dual-response switch paradigm in which we address trial-by-trial switches from dual- to single-response performance and vice versa—it is possible to derive clear and experimentally testable predictions from Gestalt theory that can be directly pitted against Structuralist accounts. Our present results are also in line with other recent evidence in favor of action Gestalten: When a certain single response [A] is more frequently practiced, this practice does not appear to transfer to allow for an easier execution of the A-part of dual [A + B] responses^50^. In addition, recent research on action imitation showed that executing a dual action (lifting both index and middle finger) is facilitated by observing a corresponding dual action but not by seeing the two composite actions (i.e., one stimulus hand lifts the index finger while another stimulus hand lifts the middle finger)^51^. Note that while our present study still involves rather basic actions, we believe that similar Gestalt representation formats occur as task complexity increases (especially in complex body movements such as dancing). In fact, Structuralist accounts typically have a hard time explaining how it is even possible that complex actions (e.g., dancing, a one-man band) evolved in the first place, as a distinct and time-consuming^10^ selection, initiation, and control of each individual component movement (of muscles and joints) in such situations would render any complex, highly synchronized movement virtually impossible. The present Gestalt account may thus offer a solution to this “complex movement paradox”.

At first sight, distinct Gestalt-like representations appear to lack parsimony: Why not benefit from partial feature overlap to more efficiently activate required action patterns? Probably, distinct representations are also characterized by the advantage of preventing unwanted conflict between task-relevant response requirements in situations involving switches between different requirements, thereby promoting resistance to interference (shielding, see^52^).

Nevertheless, despite the evidence for Gestalt representations in Experiments 1–3, the results from Experiment 4 together with other, similar data^12,13^ also show that behavior can principally be represented *flexibly* (i.e., Gestalt-like *or* in a Structuralist manner) depending on context (in particular, the degree of dimensional overlap across responses), demonstrating an astonishing extent of *representational flexibility,* in particular with respect to response coding*.* A corresponding flexibility with respect to task representations has previously been proposed in several lines of research: For example, it has been shown that one might instruct participants to represent a set of (e.g., 8) stimulus–response rules in terms of the individual, distinct (8) rules, or in terms of fewer (2) integrated, higher-order task rules^52,53^. Furthermore, mental task representations were shown to be flexibly configured across different groups: For example, younger adults were reported to rely more on internal (memory-retrieval-based) sources of information than older adults, the latter relying more on environmental cues to guide their behavior^54,55^. This type of mental flexibility, in particular with respect to action representation, should be further explored in the future as a potential source of intelligent behavior in general, as it is distinct from what is usually studied under the umbrella term “cognitive flexibility”, which rather focuses on a flexible “rewiring” of already established representations^56^. Despite this representational flexibility, however, we believe that the lack of any dimensional overlap (or other type of relation) between multiple concurrent actions (which fosters Structuralist representations) may represent the exception rather than the rule for real-life behavior, as the latter is typically guided towards a common object or person, or driven by a common overarching goal, thereby supporting the assumption of action Gestalt formation as a major principle of behavior control.

Note that the present study focuses on the mental representation of *simultaneous* action events. The mental representation of temporal event *sequences* in Gestalt psychology was studied in the context of melodies and the phi phenomenon, where the whole pattern displays characteristics that reach beyond those of the component elements (e.g., emotional expression emerging from a melody^57^; perception of continuous motion emerging from still images^58^). It would thus be interesting to follow up on our results by addressing representations of action sequences^48^. Furthermore, a Gestalt perspective on action control may also stimulate novel promising research lines. For example, compatibility phenomena (e.g., advantage of executing two “right” actions instead of a “right” and a “left” action, see Table 5) may be regarded as special cases of a “common fate”-like principle for actions (at least on a semantic level in the case of vocal actions used here), and temporal response grouping^59^ may be interpreted as a means to support (and reflect) motor Gestalten. In the future, the relation between other action-related phenomena and known individual Gestalt principles may be explored systematically (e.g., by re-conceptualizing motor learning in terms of Gestalt formation etc.). In addition, research in the domain of fundamental learning principles, where a long-lasting debate has emerged on elemental versus configural *stimulus* learning in association formation^6,7^, might benefit from a stronger focus on the potential role of configural inter-*action* associations, too^36^.

**Table 5:** Compatibility effects in Experiment 3 (all statistically significant, all ps < .002).

	Manual response (dual condition)		Vocal response (dual condition)	
	Compatible	Incompatible	Compatible	Incompatible
RT (ms)	825 (50)	1149 (104)	1110 (41)	1404 (52)
ER (%)	8.26 (3.24)	34.57 (4.73)	9.14 (5.28)	49.07 (9.53)

*SE* in parentheses. To achieve clean and clearly interpretable RT data in Experiment 3, correct vocal trials involved only trials in which solely the correct vocal response was given and this response was characterized by a clearly defined speech onset. However, some participants (especially in the difficult incompatible dual-action trials) tended to respond with vocal responses that involved self-corrections (“le… right”) or with additional vocal utterances prior to their actual response (“uhm…right”), and these types of responses were difficult to clearly disentangle. Due to these strict definitions of correct trials, vocal errors in these participants might have been severely overestimated (note that the frequency of these particular types of errors also prevented us from separately analyzing RTs in compatible vs. incompatible trials). Note, however, that an ANOVA including only participants with an occurrence of 5% (or lower) of such ambiguous vocal responses (11 participants) resulted in exactly the same overall pattern of significance for the ANOVA on error rates (including a lack of a significant effect of transition). Thus, any potential overestimation of vocal errors in some participants in Experiment 3 is unlikely to have affected the overall results. When including only participants with an occurrence of 5% or lower of ambiguous (see above) vocal responses (11 participants), vocal error rates (which may more accurately reflect actual vocal response accuracy) are as follows in Experiment 3: single condition: 2.1% (SE = 0.5), 3.4% (SE = 0.9), 6.1% (SE = 1.4) for full repetitions, partial repetitions, and switches, respectively; dual condition: 11.7% (SE = 3.3), 11.0% (SE = 2.2), 10.4% (SE = 1.9). For these 11 participants, vocal errors in compatible versus incompatible dual-action trials amounted to 2.90% (SE = 0.4) versus 19.13% (SE = 4.2), and manual errors in compatible versus incompatible dual-action trials amounted to 3.91% (SE = 1.7) versus 23.55% (SE = 5.4).

Finally, the present findings may also be relevant on a fundamental methodological level: The quest for rigorous experimental control has led many cognitive psychologists to utilize basic motor responses (i.e., key presses) as a proxy and *pars pro toto* for the study of behavioral foundations in general (atomistic approach^60^). Correspondingly, current theorizing on (multiple) action control typically takes a Structuralist approach by assuming individual mental representations corresponding to the elements that occur in an experimental trial in the lab (relevant/irrelevant stimuli, responses, effects) and turning them into mental codes with inhibitory and excitatory connections that thereby allow for some level of separation or integration (based on trials, tasks etc.)^30,41^. However, actual (mental) life does not come chopped up into trials and their elements, so that corresponding accounts therefore run the risk of vastly restricting their explanatory range to the very situation they are built upon: subjects repeatedly issuing highly restricted elementary behavior triggered by elementary stimulation in line with a set of rather arbitrary instructions. In line with this critique, the present results (which were notably based on similarly restricted trial-by-trial situations) delimitate the degree to which complex behavior can simply be analyzed in terms of its basic components (a hallmark of research methodology in cognition). This should remind us that the study of the principles underlying basic component behavior may not necessarily lead us towards a full understanding of more complex actions that actually characterize human behavior. Instead, a Gestalt view on mental action representation may provide a novel explanatory angle for understanding the human ability to display complex, temporally well-organized behavior.

## Data Availability

The datasets generated during and/or analyzed during the current study are available from the corresponding author on request.
